# Case Report: Markedly Long-Term Preservation of Pancreatic β‐Cell Function in a Subject With Elderly Onset of Type 1 Diabetes Mellitus Showing High-Titer Autoimmune Antibodies for Over 4 Years

**DOI:** 10.3389/fimmu.2021.752423

**Published:** 2021-11-25

**Authors:** Ryo Shigemoto, Takatoshi Anno, Fumiko Kawasaki, Kohei Kaku, Hideaki Kaneto

**Affiliations:** ^1^ Department of General Internal Medicine 1, Kawasaki Medical School, Okayama, Japan; ^2^ Department of Diabetes, Endocrinology and Metabolism, Kawasaki Medical School, Kurashiki, Japan

**Keywords:** type 1 diabetes mellitus, β-cell function, autoimmune antibody, anti-GAD antibody, anti-IA-2 antibody, elderly onset

## Abstract

Type 1 diabetes mellitus (T1DM) is mainly triggered by autoimmune β-cell destruction, usually leading to absolute insulin deficiency. Regarding the speed of β-cell destruction, there are large variations depending on age. In some adult cases, sufficient β-cell function is sometimes retained for a relatively long period and eventually they become dependent on insulin for survival. It is known that even in subjects with T1DM showing high titers of such antibodies, insulin secretory capacity is preserved under several conditions such as “honeymoon” period and slowly progressive T1DM (SPIDDM). Herein, we reported the acute onset T1DM subject with long-term preservation of β-cell function, although his anti-GAD antibody and anti-IA-2 antibody titers were very high for more than 4 years. This case is very important in that his β-cell function was preserved with dipeptidyl peptidase-4 inhibitor alone. This means that there are large variations in the speed of β-cell destruction in the onset of T1DM.

## Introduction

Type 1 diabetes mellitus (T1DM) is mainly triggered by autoimmune β-cell destruction, usually leading to absolute insulin deficiency, including latent autoimmune diabetes of adulthood (1). Regarding the speed of β-cell destruction, there are large variations depending on age; it is relatively rapid in children and relatively slow in adults. In some adult cases, sufficient β-cell function is sometimes retained for a relatively long period and eventually they become dependent on insulin for survival. Autoimmune markers of T1DM include islet cell autoantibodies (ICA) and autoantibodies to glutamic acid decarboxylase (GAD), insulinoma-associated protein-2 (IA-2), and zinc transporter 8 (ZnT8). It is known, however, that even in subjects with T1DM showing high titers of such antibodies, insulin secretory capacity is preserved under several conditions such as “honeymoon” period (2) and slowly progressive T1DM (SPIDDM) (3).

## Case Description

A 67-year-old man was referred to our office by his primary care physician for evaluation of hyperglycemia and elevated hemoglobin A1c (HbA1c) level. In previous yearly physical examination, his laboratory data were as follows: plasma glucose, 107 mg/dl; HbA1c, 5.6% at the age of 66. In addition, 3 months before his plasma glucose was 129 mg/dl and HbA1c was 6.8%. There was no significant past medical and family history. His height, body weight, and body mass index (BMI) were 155.0 cm, 55.0 kg, and 22.9 kg/m^2^, respectively. His vital signs were as follows: temperature, 36.7°C; blood pressure, 118/62 mmHg; heart rate, 76 beats/min; and oxygen saturation, 98%. **Table 1** shows laboratory data on admission. Diabetes-associated data were as follows: plasma glucose, 391 mg/dl; HbA1c, 13.5%; glycoalbumin, 55.3%; total ketone body, 1,195.7 μmol/L; acetoacetate, 265.5 μmol/L; and β-hydroxybuterate 930; 1 μmol/L. In addition, autoimmune markers of diabetes mellitus were as follows: anti-GAD antibody, 61,841.1 U/ml; anti-IA-2 antibody, 18 U/ml; anti-ICA, negative; and anti-ZnT8 antibody, negative.

**Table 1:** Laboratory data on admission in this subject.

Variable	Result	Reference range	Variable	Result	Reference range
**Peripheral blood**			**Diabetes marker**		
White blood cells (/μl)	3,970	3,300–8,600	Plasma glucose (mg/dl)	391	
Red blood cells (×10^4^/μl)	508	435–555	Hemoglobin A1c (%)	13.5	4.9–6.0
Hemoglobin (g/dl)	15.6	13.7–16.8	Glycoalbumin (%)	55.3	12.4–16.3
Platelets (×10^4^/μl)	17.3	15.8–34.8	Total ketone body (μmol/L)	1,195.7	0.0–130.0
**Blood biochemistry**			Acetoacetate (μmol/L)	265.6	0.0–55.0
Total protein (g/dl)	7.0	6.6–8.1	β-Hydroxybuterate (μmol/L)	930.1	0.0–85.0
Albumin (g/dl)	4.7	4.1–5.1	Insulin (μU/ml)	<1.0	1.84–12.2
Globulin (g/dl)	2.3	2.2–3.4	GAD antibody (U/ml)	61,841.1	0–4.9
Total bilirubin (mg/dl)	1.1	0.4–1.5	IA-2 antibody (U/ml)	18	0–0.3
AST (U/L)	28	13–30	ICA (JDF UNIT)	Negative	<1.25
ALT (U/L)	34	10–42	ZnT8 antibody (U/ml)	Negative	<15.0
LDH (U/L)	183	124–222	Antinuclear antibody	<40	0–39
ALP (U/L)	151	106–322	HLA-DNA typing	DRB1*09:01:02, 13:01:01	
γ-GTP (U/L)	34	13–64		DQB1*03:03:02, 06:03:01	
BUN (mg/dl)	16	8–20	**Endocrine marker**		
Creatinine (mg/dl)	0.68	0.65–1.07	ACTH (pg/ml)	56.5	7.2–63.3
Cholinesterase (U/L)	281	240–486	Cortisol (μg/dl)	16.1	6.24–18.0
Uric acid (mg/dl)	2.6	2.6–5.5	DHEA-S (μg/dl)	202	76–386
CRP (mg/dl)	0.02	<0.14	TSH (μU/ml)	2.315	0.35–4.94
BNP (pg/ml)	11.7	<18.4	Free thyroxine (ng/dl)	0.71	0.70–1.48
Sodium (mmol/L)	136	138–145	Urinary test		
Potassium (mmol/L)	4.2	3.6–4.8	Urinary pH	5.5	5.0–7.5
Chloride (mmol/L)	99	101–108	Urinary protein	–	–
**Dyslipidemia marker**			Urinary sugar	3+	–
Total cholesterol (mg/dl)	215	142–248	Urinary ketone body	1+	–
LDL cholesterol (mg/dl)	117	65–139	Urinary bilirubin	–	–
HDL cholesterol (mg/dl)	81	40–90	Urinary blood	–	–
Triglyceride (mg/dl)	74	40–149			

AST, aspartate aminotransferase; ALT, alanine aminotransferase; LDH, lactate dehydrogenase; ALP, alkaline phosphatase; γ-GTP, γ-glutamyltranspeptidase; BUN, blood urea nitrogen; CRP, C-reactive protein; BNP, brain natriuretic peptide; LDL, low-density lipoprotein; HDL, high-density lipoprotein; GAD antibody, antiglutamic acid decarboxylase; IA-2, anti-insulinoma-associated tyrosine phosphatase-like protein-2; ICA, anti-islet cell antigen; ACTH, adrenocorticotropic hormone; DHEA-S, dehydroepiandrosterone sulfate; TSH, thyroid-stimulating hormone.

**Figure 1:**
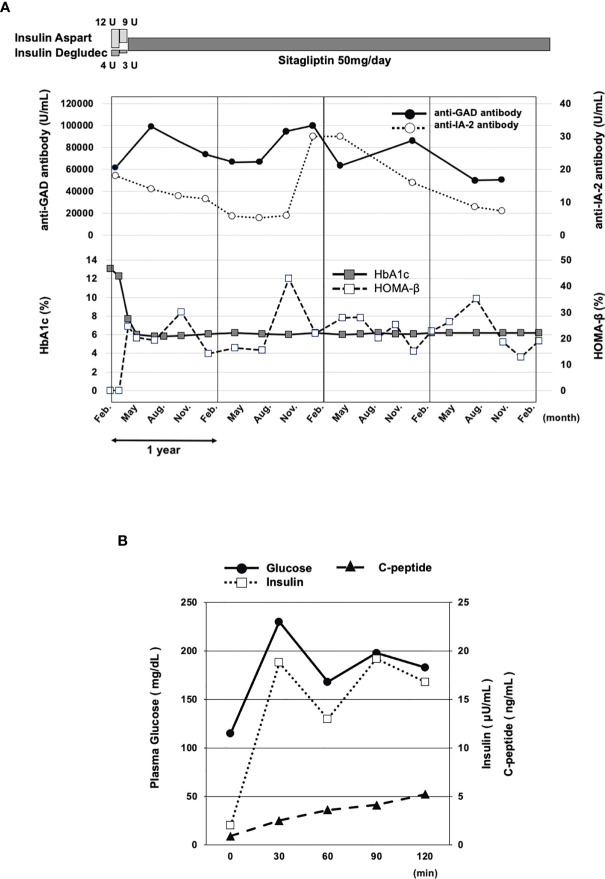
**(A)** Time course of clinical parameters in this subject. On admission, we started insulin therapy. About 3 months later, we stopped insulin therapy and changed to 50 mg of sitagliptin. His good glycemic control continued, although his anti-GAD antibody and anti-IA-2 antibody titers were very high for over 4 years. HOMA-β showed his β-cell function was retained, although it was decreased. **(B)** Seventy-five grams of oral glucose tolerance test in this subject 4 years after being diagnosed of T1DM. His insulin secretory capacity was preserved, although it was decreased. HbA1c, hemoglobin A1c; HOMA-β, homeostatic model of assessment-β; GAD, glutamic acid decarboxylase; IA-2, insulinoma-associated protein-2.

On admission, we thought that he had acute onset T1DM and started insulin therapy (4 units of aspart before each meal and 4 units of degludec at once). After, his glycemic control was dramatically improved, and he was discharged about 2 weeks later. It is difficult to decide whether we should continue or stop insulin in patients with SPIDDM. In this subject, we chose DPP-4 inhibitor instead of insulin or other medicine for the following reasons. First, a recent consensus statement on latent autoimmune diabetes in adults (LADA), which may be slightly different from SPIDDM in pathology, recommends repeated measurement of serum C-peptide levels and selection of treatments, such as insulin or antidiabetic drug, depending on each C-peptide level (4). This report suggests that DPP-4 inhibitors represent a potential therapeutic alternative for effective management of LADA. Second, while metformin is recommended to be used as a first choice in Europe and the USs, DPP-4 inhibitor is often used as a first choice in Japan. In addition, since our patient was not obese, we selected DPP-4 inhibitor, but not another antidiabetic drug such as metformin. Based on the abovementioned reasons, we tapered insulin dose in outpatient clinic. About 3 months later, we discussed the necessity of insulin therapy with our medical staffs and the patient himself, and finally we took the courage to stop insulin therapy and change to 50 mg of sitagliptin. Nonetheless, interestingly, his glycemic control was continuously very good and HbA1c levels were about 6% for over 4 years with sitagliptin therapy alone (**Figure 1A**). In addition, his homeostatic model of assessment (HOMA)-β, a marker for pancreatic β-cell function (criteria for decreased insulin secretion range: <30), showed that his β-cell function was preserved for such a long period, although it was lower compared to its reference range.

Since β-cell function in this subject was preserved for a long period (about 4 years) with dipeptidyl peptidase-4 inhibitor alone, we examined his glucose-stimulated insulin secretion (GSIS) with 75 g oral glucose tolerance test. As shown in **Figure 1B**, his GSIS was preserved, although it was decreased. HOMA-β was correlated with insulinogenic index, fasting serum C-peptide immunoreactivity index (CPI) and secretory units of islets in transplantation (SUIT) index. His insulinogenic index, CPI and SUIT index were 0.15 (criteria for decreased insulin secretion: < 0.4), 0.78 (≤0.8) and 25.33 (<30), respectively. The abovementioned all β-cell markers indicated that his β-cell function was not exhausted, although it was deteriorated to some extent.

## Discussion

Herein, we reported the acute onset T1DM subject with long-term preservation of β-cell function, although his anti-GAD antibody and anti-IA-2 antibody titers were very high for over 4 years. The Japan Diabetes Society (JDS) classifies T1DM as fulminant, acute onset, or slowly progressive T1DM based upon clinical presentation and progression (5). Acute onset T1DM is diagnosed, if diabetes ketosis or ketoacidosis occurs around <3 months after the onset of hyperglycemic symptoms. In addition, subjects with acute onset T1DM (autoimmune) need continuous insulin therapy after diabetes is diagnosed. Ketosis can be confirmed by either urine and/or serum estimation. The definition clarifies that ketosis may not occur if insulin therapy is instituted early and that a honeymoon period may occur with regard to insulin requirement. At present, it is difficult to meticulously manage T1DM conditions. The currently accepted classification of T1DM (autoimmune and idiopathic) is based only upon its etiology. Since our patient showed hyperglycemic symptoms and ketosis within 3 months, we diagnosed him as acute onset T1DM. Most interesting point in this case was that his β-cell function was preserved for a long period, although his anti-GAD antibody and anti-IA-2 antibody titers were continuously very high. This is very rare case, considering that insulin secretory capacity is usually completely exhausted, when anti-GAD antibody and anti-IA-2 antibody show such high titers.

One possibility of his pathology is that he was under a very long-term honeymoon period or partial clinical remission phase. Honeymoon period is transiently induced by significant amount of endogenous insulin production by residual β cells. During honeymoon period, patients may require progressive smaller doses of insulin for good glycemic control. In literature, honeymoon period usually appears approximately 3 months after starting insulin therapy, but the duration ranges from 1 month up to 13 years in children (6). In adults, there are no reports about duration ranges of honeymoon period and required insulin amount. Moreover, there are no reports of the honeymoon phase lasting for 4 years with oral medication alone. Another possibility of his pathology is that he had slowly progressive T1DM (SPIDDM) and his glycemic control was significantly aggravated by some stimuli such as a marked change of life style. Although high-titer autoimmune antibodies can be explained by the presence of SPIDDM, we think such possibility is quite low for the following reasons. First, according to the interview with this subject, it seemed that there was not any marked change in his eating, although he drunk over 1 L of PET bottle of juice and ate a lot of fruits. Second, while insulin secretory capacity is not usually exhausted completely by soft drink ketosis, his insulin secretory capacity was completely exhausted at the beginning for a couple of weeks. Final possibility is that his pathology is based on some unknown concept or etiology. In conventional concept, it is a common sense that stopping insulin therapy is a life-threatening performance and is recognized as one of contraindications in subjects with T1DM. Therefore, we assume that there is some new concept or etiology which could well explain the pathology of this subject, although we do not know the precise mechanism for it at present. It is difficult, however, to explain the precise mechanism why β-cell function was preserved even with high titers of autoantibodies in this subject. We are sure that this subject had T1DM because both GAD and IA-2 antibodies were positive. In addition, we think that β cells were not destroyed in this subject because insulin secretory capacity was substantially preserved. Therefore, only one point that we can say at present for sure is that insulin secretory capacity is preserved before high titers of autoantibodies lead to destroy β-cell.

## Conclusion

Taken together, we should bear in mind that β-cell function is maintained for a long period, even in subjects with acute onset T1DM. In addition, we think that this case is very important in that his β-cell function was preserved with DPP-4 inhibitor alone, although his anti-GAD antibody and anti-IA-2 antibody titers were very high for over 4 years. Therefore, it would be important to repeatedly check β-cell function even in T1DM subjects with high-titer autoimmune markers.

## Data Availability Statement

The raw data supporting the conclusions of this article will be made available by the authors, without undue reservation.

## Ethics Statement

Written informed consent was obtained from the individual(s) for the publication of any potentially identifiable images or data included in this article.

## Author Contributions

TA researched data and wrote the manuscript. RS and FK researched data and contributed to the discussion. KK and HK reviewed the manuscript. All authors contributed to the article and approved the submitted version.

## Conflict of Interest

The authors declare that the research was conducted in the absence of any commercial or financial relationships that could be construed as a potential conflict of interest.

## Publisher’s Note

All claims expressed in this article are solely those of the authors and do not necessarily represent those of their affiliated organizations, or those of the publisher, the editors and the reviewers. Any product that may be evaluated in this article, or claim that may be made by its manufacturer, is not guaranteed or endorsed by the publisher.
